# Factors Affecting Length of Stay in the Emergency Department in Patients Who Presented with Abdominal Pain

**DOI:** 10.1155/2020/5406516

**Published:** 2020-05-27

**Authors:** Ar-aishah Dadeh, Pitshaya Phunyanantakorn

**Affiliations:** Department of Emergency Medicine, Songklanagarind Hospital, Faculty of Medicine, Prince of Songkla University Hat Yai, Songkhla 90110, Thailand

## Abstract

**Objective:**

We aimed to identify factors affecting length of stay in the emergency department in patients who presented with abdominal pain.

**Methods:**

A retrospective cohort study was conducted from 1 January 2017 to 31 December 2017. The medical records were reviewed from 217 patients older than 15 years with the chief complaint of abdominal pain. The patients were divided into emergency department length of stay (EDLOS) < 4 hours and ≥4 hours. The two groups were compared in terms of baseline characteristics, physical examination, time of presenting, attending physicians, interdepartmental consultations, investigations, ED disposition, final diagnosis, and mortality. The significant factors affecting longer EDLOS were examined using univariate and multivariate analyses by logistic regression.

**Results:**

Factors affecting longer EDLOS were age ≥50 (odds ratio (OR) 3.17, 95% confidence interval (CI) 1.36–7.42), interdepartmental consultation ≥2 specialists (OR 71.82, 95% CI 5.67–909.51), blood testing ≥2 rounds (OR 85.6, 95% CI 4.22–1734.6), and ultrasonography (OR 8.28, 95% CI 1.84–37.26).

**Conclusion:**

The study found that the statistically significant factors that prolonged EDLOS in patients with the chief complaint of abdominal pain were age, rounds of blood test, interdepartmental consultation, and the need for ultrasonography.

## 1. Background

Overcrowding in the emergency department (ED) is a worldwide problem including the United States of America, Europe, and Asia. The American College of Emergency Physicians stated that crowding occurs when the identified need for emergency services exceeds available resources for patient care in the ED, hospital, or both. Overcrowding reduces the effective capacity of the management and treatment of critically ill patients in the ED [1]. ED length of stay (EDLOS) directly affects ED overcrowding and is a key indicator to monitor ED performance [2]. Longer durations of EDLOS were reported to be associated with the rates of hospital admission, 10-day mortality, and dissatisfaction [2].

The National Health Service of the United Kingdom announced its intention to improve the quality of ED care by instituting a maximum length of ED stay of 4 hours [3]. Patients who spent more than 4 hours in the ED were less satisfied with their environment than those who spent less than 4 hours [4].

In the year 2017, the ED of Songklanagarind Hospital had 40,150 visits. The number of patients who spent >4 hours in the ED was 6,800 (17%). Abdominal pain was the leading presenting symptom among these patients which accounted for 31%. Prolonged EDLOS in a patient with abdominal pain delays a patient's definitive treatment or surgery leading to morbidity and mortality.

The aim of this study was to identify factors affecting length of stay in the ED in patients who presented with abdominal pain.

## 2. Materials and Methods

### 2.1. Study Design and Setting

A retrospective cohort study was conducted in the ED of Songklanagarind Hospital which is a teaching hospital and a tertiary care medical center with a capacity of 850 beds. Our emergency department is staffed by 1-2 interns, 4-5 emergency medicine residents, one board certified emergency medicine physician as a consultant who attends each shift, and 8–10 round-the-clock registered nurses per shift. Data were collected from January 2017 to December 2017. Patients older than 18 years with the chief complaint of abdominal pain were enrolled. We excluded trauma patients, pregnant patients, patients who were referred from other hospitals, and patients who had incomplete data.

All patients who presented to the ED were categorized into the five-level triage system using the Emergency Severity Index (ESI). The triage protocol used in the ED to facilitate the prioritization of patients was based on the urgency of treatment upon the patient's conditions and resources needed [5]. Emergency medicine residents obtained the patient's history and performed the physical examination with the assistance of medical personnel. Some patients required further investigations such as blood tests, plain film, ultrasound, or a computed tomography (CT) scan to make the diagnosis. After the diagnosis was made, the emergency physician consulted a specialist for definitive treatment if indicated. Afterward, the patients were discharged home, admitted to the ED observational unit (EDOU), admitted to the ward or ICU, or referred to other hospitals.

### 2.2. Data Collection

The data collected from the electronic medical records included baseline characteristics, comorbid diseases, pain characteristics, type of insurance, route of presentation, severity triage, time of presenting, attending physician, interdepartmental consultation, laboratory investigations, imaging investigations, EDLOS, ED disposition, mortality, and final diagnosis. The patients were categorized into either the EDLOS group of ≥4 hours or the EDLOS group of <4 hours.

### 2.3. Outcome Measurements

The primary outcome was to identify the factors affecting the EDLOS in patients who presented with abdominal pain. Secondary outcomes were the final diagnosis and to determine the associations between EDLOS and 24-hour, 7-day, and 28-day mortality.

### 2.4. Statistical Analysis

The study population sample size was calculated using the n4Studies program to test two independent proportions. A total of 208 EDLOS patients were enrolled: 52 in the ≥4-hour group and 156 in the <4-hour group. The confidence level was set at 95%, and the power was 80%.

All data were entered into EpiData Manager (version 4.4.2.1), and the statistical analysis was conducted using R software (version 3.5.1). Continuous variables are presented as mean and median. Discrete variables are presented as percentage. A comparison between the continuous variables in the two groups used the Student's *t*-test or Wilcoxon rank-sum test. Comparison between discrete variables in the two groups used the Pearson's chi-square test or Fisher's exact test. Significant factors associated with EDLOS ≥4 hours (*P* < 0.2) identified during univariate logistic regression analysis were introduced into a subsequent multivariate logistic regression analysis. Analytical results are described as odds ratio (OR) with 95% confidence interval (CI). A *P* value less than 0.05 was considered statistically significant.

### 2.5. Compliance with Ethical Requirements

The ethics committee of Prince of Songkla University approved this study. The institutional review board of Prince of Songkla University is affiliated with the International Conference on Harmonization in Good Clinical Practice. According to our institutional review board protocol for waiver of informed consent, the requirement for consent was waived because the participants had no more than minimal risk and the patients received standard treatment procedures. All research information was kept as confidential data in an encrypted file with password and limited data access by only the researcher and assistant.

## 3. Results

### 3.1. Demographic Data

During the study period, 3,794 patients with a chief complaint of abdominal pain were registered. A computer-based randomization system selected 217 patients who met the enrollment criteria. The baseline characteristics of the patients are shown in Table 1. Patients who spent ≥4 hours in the ED were older (median 58 years) than those who spent <4 hours in the ED (median 48 years).

### 3.2. Predictive Factors for EDLOS

There were no significant differences in body temperature, pain score, or location of pain between the two groups (Table 2). Also, no significant differences were identified in times of presenting or attending physicians between the two groups. On the other hand, patients who required ≥2 interdepartmental consultations tended to spend >4 hours in the ED (16.4% (≥4 hours) vs 0.6% (<4 hours)) (Table 3). Additional factors associated with EDLOS ≥4 hours versus <4 hours were the requirement for ultrasonography (23.6% vs 1.9%), CT scan (23% vs 2.5%), and ≥2 rounds of blood testing (18.2% vs 3.1%).

### 3.3. Patient Outcome and Mortality

The percentages of patients admitted to the hospital wards, ICU, EDOU, or referred to other hospitals in the EDLOS ≥4-hour group were 56.4%, 3.6%, 7.3%, and 7.1%, respectively, whereas the percentages in the EDLOS <4-hour group were 24.7%, 0%, 1.9%, and 3.1%, respectively (Table 4). The percentages of patients who spent ≥4 hours with a definite diagnosis of either acute cholecystitis or acute pancreatitis were 10.9% and 12.7%, respectively. However, in the group of EDLOS <4 hours, none of the cases had acute cholecystitis and only 1.2% had acute pancreatitis (Table 5). Four patients in the EDLOS ≥4-hour group with diagnoses of intestinal obstruction, ruptured hepatoma, psoas abscess with salmonella septicemia, and acute pancreatitis died within or at 7 days after hospital admission (Table 6). Multivariate analysis revealed that the significantly associated factors for EDLOS ≥4 hours were age ≥50 years old, interdepartmental consultation ≥2 specialists, blood testing ≥2 rounds, or the requirement for ultrasonography (Table 7).

## 4. Discussion

The statistically significant factors associated with EDLOS ≥4 hours after the multivariate logistic regression analysis were age, rounds of blood testing, interdepartmental consultation, and the need for ultrasonography. Elderly patients with the chief complaint of abdominal pain were likely to stay in the ED longer because of multiple chronic diseases, exceptional physiology leading to an atypical presentation with delayed symptoms, unreliable vital signs in response to disease, and unpredictable physical examination. Hence, they required additional investigations, imaging, and consultation services [6].

Multiple rounds of blood testing were strongly associated with longer length of stay. The adjusted OR values were higher in those who had ≥2 rounds of blood testing. The results of the present study were compatible with the studies of Wibulpolpresert et al. [7], Gardner et al. [8], and Henneman et al. [9]. Those studies reported that more diagnostic testing was associated with longer EDLOS. However, a study by Walsh showed that blood test results had a minimal impact on the expected patient disposition from the ED. This was likely due to dynamic changes in the clinical conditions of the patients which required multiple rounds of blood tests. Also, inexperienced physicians may be a factor. Physicians may need to exercise caution when requesting blood tests. Bedside blood testing, such as troponin level, should be available to use in high-risk patients with epigastric pain where acute coronary syndrome cannot be ruled out.

Interdepartmental consultation is a common and important aspect of ED practice which can lead to delays in patient flow. There are different types of consultations: consultations for admission; for opinion only; for special procedures; for transfer of care; and for outpatient referrals [10]. Delays can also be caused by poor communication between ER physicians, and consultants, an insufficient number of consultation staff personnel, and a shortage of in-patient beds. A study by Cho et al. [11] found a decreased ED length of stay by implementation of a computerized consultation management system in a tertiary care teaching hospital. The automated consultation and monitoring process formalized communication between physicians providing ED patient care in the academic ED with high consultation and admission rates.

Ultrasonography is used mainly in the initial diagnosis of acute cholecystitis and acute appendicitis. Hence, patients with a diagnosis of acute cholecystitis tended to spend more time in the ED. Ultrasonography services in Songklanagarind Hospital are performed at the Department of Radiology by radiologists which causes delays due to the transport time and waiting times for an official radiology report. Thamburaj and Sivitz [12] found that ED bedside ultrasound by trained emergency medicine physicians produced a significant reduction in length of stay in the ED, regardless of the availability of a radiology ultrasound technologist.

Two definite diagnoses associated with longer EDLOS were acute cholecystitis and acute pancreatitis. Diagnostic imaging, such as ultrasonography and CT scan, is required in the diagnosis of acute cholecystitis. The imaging process took time which resulted in longer durations of EDLOS. Acute pancreatitis is a medical gastrointestinal condition which requires admission. Since Songklanagarind Hospital is a tertiary care hospital with a limited number of in-patient beds, the patients had to wait longer in the ED for bed availability.

Four patients who had an EDLOS of ≥4 hours died within or at 7 days after admission. Furthermore, we found that the two ICU admissions spent longer than four hours in the ED for resuscitation, investigations, and consultation purposes. The patient who spent the longest time in the ED was elderly who suffered from septic shock caused by salmonella septicemia with psoas abscess with multiple organ dysfunction. A previous study showed that clinical outcomes were not significantly different between early and delayed ICU admissions in sepsis patients. [13] In contrast, Zhang et al. showed increased in-hospital mortality when sepsis patients requiring ICU admission stayed longer than 12 hours in the ED [14]. Several reasons may cause delayed ICU admissions including delayed decision by the ED physician, shortage of ICU beds, waiting for radiologic examination, and ED crowding.

Two patients with diagnoses of ruptured hepatoma and acute pancreatitis had longer EDLOS durations because of interdepartmental consultations which took almost 4 hours. The patient with a diagnosis of acute pancreatitis stayed longer in the ED because of a shortage of in-patient beds. Those factors that prolonged the EDLOS should be considered and modified to reduce the mortality rate.

Fourteen patients (25.5%) of the discharged group spent longer than four hours in the ED. In this group, four patients needed one specialist consultation and one patient needed two specialist consultations. Five patients required more than one round of blood testing. One study showed that prolonged ED stays for admitted patients were associated with prolonged throughput times for patients discharged home from the ED [15]. One large study that involved 995,379 ED visits found that periods of high ED crowding were associated with increased inpatient mortality and modest increases in length of stay and costs for admitted patients [1]. Although the group of discharged patients who spent a longer time in the ED possibly caused crowding, the EDLOS of the admitted patients was not directly affected. However, poor patient outcomes were possibly associated with ED overcrowding. A further study on the impact of EDLOS of discharged patients on admitted patient outcome would be interesting.

The present study identified organizational factors in the ED that resulted in longer lengths of stay. However, some other factors, such as the number of patients per shift and the National Emergency Department Overcrowding Score, should be considered as well. We must acknowledge the limitations of the present study. The study was retrospective in nature which was conducted in a single ED and some data may be missing.

## 5. Conclusions

The study found that the statistically significant factors that prolonged the EDLOS in patients with the chief complaint of abdominal pain were age, rounds of blood tests, interdepartmental consultation, and the need for ultrasonography. Hence, the emergency physician should remain aware of the time needed to perform blood and imaging tests and be skillful in bedside sonography to shorten the EDLOS.

## Figures and Tables

**Table 1:** Baseline characteristics of the patients.

Characteristic	EDLOS < 4 hours (*n* = 162)	EDLOS ≥ 4 hours (*n* = 55)	*P* value
Age, years, median (IQR)	48 (27, 64.8)	60 (44, 68)	0.013
Male	62 (38.3)	29 (52.7)	0.161

*Comorbidity*			
Diabetic mellitus	21 (13)	6 (10.9)	0.871
Hypertension	33 (20.4)	15 (27.3)	0.38
Hyperlipidemia	24 (14.8)	9 (16.4)	0.953
Malignancy	16 (9.9)	10 (18.2)	0.229
HIV infection	0 (0)	2 (3.6)	0.063
Cerebrovascular disease	6 (3.7)	5 (9.1)	0.278
Ischemic heart disease	6 (3.7)	3 (5.5)	0.696
Chronic kidney disease	3 (1.9)	3 (5.5)	0.172

*Type of insurance*			
Self-pay	64 (39.5)	14 (25.5)	0.116
Government officer	70 (43.2)	27 (49.1)	
Social security scheme	5 (3.1)	5 (9.1)	
Universal health care	23 (14.2)	9 (16.4)	

*Route of presentation*			
Self	159 (98.1)	54 (98.2)	0.692
Emergency medical services	2 (1.2)	0 (0)	
First responder	1 (0.6)	1 (1.8)	

*Note*. Data are presented as *n* (%) unless indicated otherwise. EDLOS, emergency department length of stay; IQR, interquartile range; HIV, human immunodeficiency virus.

**Table 2:** Comparison of clinical presentation and physical examination.

Characteristic	EDLOS < 4 hours (*n* = 162)	EDLOS ≥ 4 hours (*n* = 55)	*P* value
BT ≥ 37.8°C	16 (9.9)	10 (18.2)	0.162
Pain score, median (IQR)	7 (6.9)	7 (5.8)	0.161

*Locations of pain*			
Generalized	24 (14.8)	11 (20)	0.665
Epigastrium	27 (16.7)	14 (25.5)	
RUQ	30 (18.5)	10 (18.2)	
LUQ	5 (3.1)	2 (3.6)	
LLQ	21 (13)	5 (9.1)	
RLQ	32 (19.8)	6 (10.9)	
Suprapubic	14 (8.6)	5 (9.1)	
Paraumbilical	8 (4.9)	3 (5.5)	

*Note*. Data are presented as *n* (%) unless indicated otherwise. EDLOS, emergency department length of stay; BT, body temperature; RUQ, right upper quadrant; LUQ, left upper quadrant; LLQ, left lower quadrant; RLQ, right lower quadrant.

**Table 3:** Comparison of other factors.

Characteristic	EDLOS < 4 hours (*n* = 162)	EDLOS ≥4 hours (*n* = 55)	*P* value
*Times of presenting*			0.088
08:00–16:00 (morning shift)	49 (30.2)	27 (49.1)	
16:01–24:00 (afternoon shift)	74 (45.7)	18 (32.7)	
00:01–08:00 (night shift)	39 (24.1)	10 (18.1)	

*Attending physicians*			
Intern	81 (50)	26 (47.3)	0.892
Resident 1	42 (25.9)	16 (29.1)	
Resident 2	22 (13.6)	8 (14.5)	
Resident 3	13 (8)	5 (9.1)	
Staff	4 (2.5)	0(0)	

*Consultation*			<0.001
None	112 (69.1)	10 (18.2)	
1 specialist	49 (30.2)	36 (65.5)	
≥2 specialists	1 (0.6)	9 (16.4)	

*Note*. Data are presented as *n* (%). EDLOS, emergency department length of stay; IQR, interquartile range.

**Table 4:** Comparison of patient investigations and dispositions.

Characteristic	EDLOS < 4 hours (*n* = 162)	EDLOS ≥ 4 hours (*n* = 55)	*P* value
Film KUB	14 (8.6)	2 (3.6)	0.369
Film acute abdominal series	32 (19.8)	16 (29.1)	0.103
Ultrasonography	3 (1.9)	13 (23.6)	<0.001
Computed tomography	4 (2.5)	13 (23.6)	<0.001

*Blood testing*			<0.001
None	50 (30.9)	1 (1.8)	
1 round	107 (66)	44 (80)	
≥2 rounds	5 (3.1)	10 (18.2)	

*ED disposition*			
Discharge	114 (70.4)	14 (25.5)	<0.001
Admitted to ward	40 (24.7)	31 (56.4)	
Admitted to ICU	0 (0)	2 (3.6)	
Admitted to EDOU	3 (1.9)	4 (7.3)	
Referred	5 (3.1)	4 (7.1)	

*Note*. Data are presented as *n* (%). EDLOS, emergency department length of stay; KUB, kidney ureter and bladder; ED, emergency department; ICU, intensive care unit; EDOU, emergency department observation unit.

**Table 5:** Comparison of patient diagnosis and mortality.

Characteristic	EDLOS < 4 hours (*n* = 162)	EDLOS ≥ 4 hours (*n* = 55)	*P* value
Definite diagnosis			
Hepatobiliary tract diseases			
Acute cholecystitis	0 (0)	6 (10.9)	<0.001
Acute pancreatitis	2 (1.2)	7 (12.7)	0.001
Acute cholangitis	3 (1.9)	1 (1.8)	1.000
Surgical GI conditions			
Acute appendicitis	15 (9.3)	4 (7.3)	0.787
Intestinal obstruction	6 (3.7)	5 (9.1)	0.151
Perforation of intestine	4 (2.5)	1 (1.8)	1.000
Acute peritonitis	0 (0)	2 (3.6)	0.063
Medical GI conditions			
Peptic ulcer disease	15 (9.3)	4 (7.3)	0.787
Diverticular of intestine	2 (1.2)	0 (0)	1.000
Acute gastroenteritis	13 (8)	1 (1.8)	0.125
Constipation	8 (4.9)	0 (0)	0.207
Genitourinary conditions			
Urolithiasis	21 (13)	1 (1.8)	0.035
Gynecological conditions			
Pelvic inflammatory disease	4 (2.5)	2 (3.6)	0.645
Ovarian cyst with complication	3 (1.9)	3 (5.5)	0.645
Cardiovascular conditions			
Thoracoabdominal aortic aneurysm	3 (1.9)	1 (1.8)	1.000
Other conditions			
Nonspecific abdominal pain	23 (14.2)	6 (10.9)	0.697
Tumor pain	5 (3.1)	1 (1.8)	1.000
Mortality			0.004
Survived	162 (100)	51 (92.7)	0.004
Dead in 7 days	0 (0)	3 (5.5)	
Dead in 8–28 days	0 (0)	1 (1.8)	

*Note*. Data are presented as *n* (%). EDLOS, emergency department length of stay; GI, gastrointestinal.

**Table 6:** Summary of patient mortality in 7 days after admission.

No.	Diagnosis	EDLOS	Mortality after admission
1	Psoas abscess with salmonella septicemia	12 hours 14 minutes	25 hours
2	Ruptured hepatoma	4 hours 10 minutes	5 days
3	Acute pancreatitis	5 hours 40 minutes	7 days
4	Intestinal obstruction	5 hours	7 days

EDLOS, emergency department length of stay.

**Table 7:** Multivariate comparison of factors.

Variables	Unadjusted OR (95% CI)	Adjusted OR (95% CI)	*P* value
Age ≥50	1.88 (1–3.54)	3.17 (1.36–7.42)	0.006
*Consultation*			
None	1	1	<0.001
Specialist	8.23 (3.78–17.89)	4.3 (1.75–10.59)	
≥2 specialists	100.8 (11.57–878.33)	71.82 (5.67–909.51)	
*Blood testing*			
None	1	1	<0.001
1 round	20.56 (2.75–153.48)	19.58 (1.21–317.97)	
≥2 rounds	100 (10.52–950.39)	85.6 (4.22–1734.6)	
*Ultrasonography*	16.4 (4.47–60.23)	8.28 (1.84–37.26)	

OR, odds ratio; CI, confidence interval.

## Data Availability

The retrospective data used to support the findings of this study are available from the corresponding author upon request.

## References

[1] Sun B. C., Hsia R. Y., Weiss R. E. (2013). Effect of emergency department crowding on outcomes of admitted patients. *Annals of Emergency Medicine*.

[2] Richardson D. B. (2006). Increase in patient mortality at 10 days associated with emergency department overcrowding. *Medical Journal of Australia*.

[3] Department of Health (2000). *The NHS Plan: A Plan for Investment—A Plan for Reform*.

[4] Walsh M., Knott J. C. (2010). Satisfaction with the emergency department environment decreases with length of stay. *Emergency Medicine Journal*.

[5] Gilboy N., Tanabe T., Travers D., Rosenau A. M. (2012). *Emergency Severity Index: A Triage Tool for Emergency Department Care—Version 4 Implementation Handbook*.

[6] Spangler R., Phum T. V., Khoujah D., Martinez J. P. (2014). Abdominal emergencies in the geriatric patient. *International Journal of Emergency Medicine*.

[7] Wibulpolprasert A., Sittichanbuncha Y., Sricharoen P., Borwornsrisuk S., Sawanyawisuth K. (2014). Factors associated with overcrowded emergency rooms in Thailand: a medical school setting. *Emergency Medicine International*.

[8] Gardner R. L., Sarkar U., Maselli J. H., Gonzales R. (2007). Factors associated with longer ED lengths of stay. *The American Journal of Emergency Medicine*.

[9] Henneman P. L., Nathanson B. H., Li H. (2010). Emergency department patients who stay more than 6 hours contribute to crowding. *The Journal of Emergency Medicine*.

[10] Lee R. S., Woods R., Bullard M., Holroyd B. R., Rowe B. H. (2008). Consultations in the emergency department: a systematic review of the literature. *Emergency Medicine Journal*.

[11] Cho S. J., Jeong J., Han S. (2011). Decreased emergency department length of stay by application of a computerized consultation management system. *Academic Emergency Medicine*.

[12] Thamburaj R., Sivitz A. (2013). Does the use of bedside pelvic ultrasound decrease length of stay in the emergency department?. *Pediatric Emergency Care*.

[13] Agustin M., Price L. L., Andoh-Duku A., La Camera P. (2017). Impact of delayed admission to the intensive care unit from the emergency department upon sepsis outcomes and sepsis protocol compliance. *Critical Care Research and Practice*.

[14] Zhang Z., Bokhari F., Guo Y., Goyal H. (2019). Prolonged length of stay in the emergency department and increased risk of hospital mortality in patients with sepsis requiring ICU admission. *Emergency Medicine Journal*.

[15] Krall S. P., Guardiola J., Richman P. B. (2016). Increased door to admission time is associated with prolonged throughput for ED patients discharged home. *The American Journal of Emergency Medicine*.

